# Effects of plant essential oil supplementation on growth performance, immune function and antioxidant activities in weaned pigs

**DOI:** 10.1186/s12944-018-0788-3

**Published:** 2018-06-15

**Authors:** Guoqi Su, Xuanwu Zhou, Yu Wang, Daiwen Chen, Guang Chen, Yan Li, Jun He

**Affiliations:** 1 Institute of Animal Nutrition, Sichuan Agricultural University, Chengdu, Sichuan 611130 People’s Republic of China; 2 Cheng Du Hua Luo Bio-Tech Col., Ltd, Chengdu, Sichuan 610062 People’s Republic of China

**Keywords:** Antioxidant activities, Growth performance, Immunity, Plant essential oil, Weaned pigs

## Abstract

**Background:**

The aim of this study was to determine the effects of plant essential oil supplementation on growth performance, immune function and antioxidant activities in weaned pigs.

**Methods:**

In the study, 24 weaned pigs were used to explore the effects of plant essential oil (PEO) on growth performance, immune properties and antioxidant activities. Pigs were fed with a basal diet (CON) or basal diet containing different concentrations of PEO (PEO50: 50 ppm; PEO100: 100 ppm; PEO200: 200 ppm). After 3 weeks, all pigs were slaughtered and blood and tissue samples were collected for biochemical analysis.

**Results:**

The results showed that PEO supplementation quadratically increased body weight gain (BWG) (*P* = 0.031), linearly (*P* <  0.05) and quadratically (*P* <  0.05) decreased F:G. In addition, IgG increased linearly (*P* <  0.05) and IgM increased linearly (*P* <  0.05) and quadratically (*P* < 0.05) as PEO supplementation. Similarly, MDA in serum, jejunal mucosa and pancreas were linearly decreased (*P* < 0.05) and GSH in serum (linear and quadratic, *P* < 0.05), duodenal mucosa (linear and quadratic, *P* < 0.05) and in ileal mucosa (linear and quadratic, *P* < 0.05) were notably increased. Futhermore, antioxidant-related genes expression levels of *GST* in spleen (linear and quadratic, *P* < 0.05), *GPX*1 (quadratic, *P* < 0.05) and *SOD*1 (linear, *P* < 0.05) in spleen and GST in liver (quadratic, *P* < 0.05) were markedly upregulated by PEO supplementation increasing.

**Conclusions:**

These results suggest that PEO improves growth performance, immune function, and antioxidant activities in weaned pigs, and it may also relieve weaning stress if used as a feed additive in the livestock industry. And that supplementation 200 ppm PEO in diet would seem to be economically feasible.

## Background

Recent studies have indicated that weaning can induce oxidative stress in pigs, resulting in oxidative damage [1]. Weaning stress has been reported to disrupt intestinal health, cause diarrhea, and to reduce growth and immunity in pigs [2–4]. Over the past number of decades, antibiotics, zinc oxide, and copper sulfate have been widely utilized in the swine industries for their effects in reducing diarrhea and improving immunity in weanling pigs [5–7]. However, the abuse of these additives has led to antibiotic resistance and heavy metal residues in livestock products. The rising incidence of these serious problems has compelled research institutes and farmers alike to search for safe feed additives [8–10].

Many essential oils from plants, either extracted or in their natural form, are used for their antioxidative properties, which are mainly due to phenolic compounds in the oil or in other phytochemical fractions [11, 12]. Some nonphenolic substances also exhibit considerable antimicrobial and antioxidative potential [13, 14]. Previous studies have reported that essential oils may improve nutrient digestibility, as well as intestinal morphology and microflora [15–17]. Other studies have reported that essential oil supplementation improved the nutritional value and oxidative stability of fat, meat, and eggs, resulting in longer shelf-life [18–23]. These previous findings regarding animal products led us to hypothesize whether essential oils could improve systemic redox balance and reduce oxidative injury induced by weaning stress in young pigs.

**Fig. 1:**
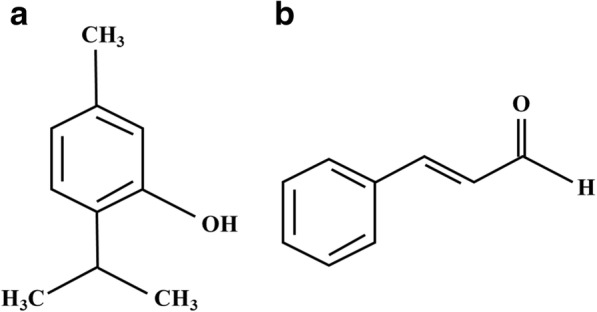
Chemical structure of thymol (**a**) and cinnamaldehyde (**b**)

Cinnamaldehyde is an organic compound with the formula C_6_H_5_CH=CHCHO, and thymol is a natural monoterpene phenol with the formula C_10_H_14_O (Fig. 1). Although several studies have indicated that cinnamaldehyde and thymol can improve growth performance, nutrient digestibility, and intestinal morphology, and stabilize the microflora of weaned pigs and poultry [11, 17, 24, 25], the effects of cinnamaldehyde and thymol on antioxidant activity and immune function in weaned pigs is still unclear. Therefore, the purpose of this study was to explore the effects of dietary supplementation with plant essential oil on both immune function and antioxidant activity in weaned pigs.

## Methods

### Animals and experimental design

A total of twenty-four commercial crossbred DLY (Duroc × Large White × Landrace) male pigs weaned at 35 d with average weight 9.19 kg (SEM 0.34) were randomly assigned to four groups (*n* = 6): 1) CON (control; a basal diet), 2) PEO50 (basal diet containing 50 ppm PEO), 3) PEO100 (basal diet containing 100 ppm PEO), 4) PEO200 (basal diet containing 200 ppm PEO). PEO was provided by Cheng Du Hua Luo Bio-Tech Col. and the active ingredient of PEO was 13.5% thymol and 4.5% cinnamyl aldehyde, carrier was dextrin.

### Diets and feeding management

Diets were corn-soybean based diets and formulated according to National Research Council 2012 requirements [26]. Ingredients and nutrient composition of experimental diets are shown in Table 1. Diets were fed in mash form throughout the experiment.

**Table 1:** Compositions and nutrient levels of the basal diet (air-dried basis)^a^

Composition		Nutrient level	
Ingredients	Proportion, %	Items	Cotent, %
Maize	30.00	CP	19.80
Extruded maize	26.00	Ca	0.91
Soyabean meal, dehulled	10.50	Total P	0.55
Puffed soyabean	4.53	P (available)	0.37
Fishmeal	4.90	Lys	1.41
Soya protein concentrate	8.00	Met	0.47
Whey powder	8.00	Thr	0.79
Glucose	3.00	Trp	0.22
Soya oil	2.50	DE	14.02
Calcium carbonate	1.20		
Calcium phosphate	0.20		
L-Lys HCl	0.38		
NaCl	0.25		
Choline chloride	0.10		
DL-Met	0.15		
Trp	0.01		
L-Thr	0.03		
Vitamins^b^	0.05		
Minerals^c^	0.20		

^a^Dietary nutrient level value was analyzed

^b^The vitamin and mineral premix (maize powder as diluent) provided the following amounts per kg complete diet: retinol, 8.4 mg; cholecalciferol, 0.008 mg; vitamin E, 20 mg; menadione, 1 mg; vitamin B12, 0.03 mg; riboflavin, 5 mg; niacin, 20 mg; pantothenic acid, 15 mg; folic acid, 0.5 mg; thiamin, 1.5 mg; pyridoxine, 2 mg; biotin, 0.1 mg

^c^The mineral premix provided the following amounts per kg complete diet: Fe, 100 mg (FeSO_4_·7H_2_O); Cu, 6 mg (CuSO_4_·5H_2_O); Zn, 100 mg (ZnSO_4_·7H_2_O); Mn, 4 mg (MnSO_4_.H_2_O); Se, 0.3 mg (Na_2_SeO_3_·5H_2_O); I, 0.14 mg (KI)

The experiment was carried out at the Research Base of the Institute of Animal Nutrition of Sichuan Agricultural University. All the pigs were housed in an environmentally controlled nursery room in individual metabolism cages (1.5 m × 0.7 m × 1.0 m). The pigs had free access to diet and water throughout the 2-week feeding trial, and were fed with the experimental diets 4 times daily at 08:00, 12:00, 16:00 and 20:00. Temperature was gradually reduced from 28 °C to 23 °C, and the humidity was controlled between 50 and 60%. Pigs were weighed at 08:00 on an empty stomach on days 0 and 14.

### Sample collection

Blood samples were collected from the portal vein precava into Vacuum pick blood vessels without anticoagulation (Axygen Biotechnology Co. Ltd) at 8:00 in the morning on day 14. From each sample, serum were collected by centrifuging the blood (3500 g, 4 °C, 10 min) and immediately stored at − 20 °C. All pigs were slaughtered by exsanguination according to protocols approved by the Sichuan Agricultural University Animal Care Advisory Committee. Mucosa of duodenum, jejunum and ileum, spleen, liver and pancreas were collected and snap frozen in liquid N_2_ and then stored at − 80 °C for assay.

### Growth performance

Individual initial and final body weight were recorded on day 0 and day 14. Body weight gain (BWG) was calculated as final body weight subtract initial body weight. Average daily gain (ADG) was calculated as weight (kg)/number of days from initial to final weight.

### Immunity parameters

The levels of total protein (TP), albumin (ALB), IgG and IgM in serum were examined using the Hitachi 7020 Automatic Analyzer (Tokyo, Japan). The level of IgA in serum was measured using an ELISA test kit (R&D Systems, Minneapolis, MN, USA) according to the manufacturer’s instructions, the absorbance was determined with a 96-well microtiter plate reader Spectramax M2 (Molecular Devices, Sunnyvale, CA).

### Antioxidant indices in serum and tissues

Tissues of duodenum, jejunum, ileum, spleen, liver and pancreas were homogenized (1:10, w:v) in glass homogenizer with ice-cold 0.9% normal saline. Homogenate were collected by centrifuging 3500 g, 4 °C, 15 min. TP, glutathione (GSH), catalase (CAT), total antioxidant capacity (T-AOC), Malondialdehyde (MDA) and total superoxide dismutase (T-SOD) of serum and tissues were quantified by spectrophotometric methods using a spectrophotometer (Leng Guang SFZ1606017568, Shanghai, China).

#### Total protein concentration analysis

Total protein assay kit BCA (A045–3) was purchased from Nanjing Jiancheng Bioengineering Institute (Nanjing, China). Apple green BCA reacted with protein at 37 °C for 30 min and formed a purple compound featuring absorbance at 562 nm. TP results are expressed as aprot per litre (gprot/L) of tissues.

#### GSH content analysis

GSH content was measured according to previous report. [4] Reduced GSH assay kit (A006–2, Nanjing Jiancheng Bioengineering Institute (Nanjing, China)) was used according manufacturer’s instructions. GSH content was expressed in gGSH/L and mgGSH/gprot in serum and tissues respectively by using commercial GSH as a standard.

#### CAT activity analysis

CAT activity was measured according to previous report [27]. CAT assay kit (A007–1, Nanjing Jiancheng Bioengineering Institute (Nanjing, China)) was used according manufacturer’s instructions. The reaction of CAT decomposing H_2_O_2_ was rapidly stopped reaction after adding ammonium molybdate. The rest of H_2_O_2_ reacted with ammonium molybdate and formed a pale yellow complex compound featuring absorbance at 405 nm. CAT results were expressed in U/mL and U/gprot in serum and tissues respectively.

#### T-AOC activity analysis

T-AOC activity was measured according to previous report [28]. Total antioxidant capacity assay kit (A015–1, Nanjing Jiancheng Bioengineering Institute (Nanjing, China)) was used according manufacturer’s instructions. T-AOC activity was expressed in U/mL and U/gprot in serum and tissues respectively.

#### MDA content analysis

MDA content was analyzed as described by previous report [29]. MDA assay kit (TBA method) (A003–1, Nanjing Jiancheng Bioengineering Institute (Nanjing, China)) was used according manufacturer’s instructions. MDA reacted with thiobarbituric acid (TBA) formed a red-complex compound featuring absorbance at 532 nm. MDA results were expressed in nmol/mL in serum and tissues.

#### T-SOD activity analysis

T-SOD activity was analyzed as described by previous report [30]. Total Superoxide Dismutase (T-SOD) assay kit (Hydroxylamine method) (A001–1, Nanjing Jiancheng Bioengineering Institute (Nanjing, China)) was used according manufacturer’s instructions. Superoxide ions reacted with 2-(4-iodophenyl)-3- (4-nitrophenol)-5-phenyltetrazolium chloride to form a red formazan dye featuring absorbance at 550 nm. T-SOD activity was expressed in U/mL in serum and tissues.

### RNA isolation and real-time quantitative PCR

Total RNA from samples of spleen, liver and pancreas was extracted using TRIzol reagent (TaKaRa, Dalian, China) according to the manufacturer’s instructions. RNA concentration was measured by Nanodrop 2000 (Thermo Fisher Scientific, Wilmington,DE, USA). The integrity of RNA was verified by eletrophoretic analysis. Complementary DNA (cDNA) was achieved by reverse transcription with 2 μg RNA sample using the PrimeScript™ RT reagent Kit (TaKaRa, Dalian, China) according to the manufacturer’s instructions. 20 μL final reaction volume of cDNA was then diluted to 250 μL using nuclease-free water and stored at − 20 °C. The cDNA was used as the template for PCR. Real-time quantitative PCR was performed on cDNA using the ABI PRISM 7500 Fast Sequence Detection System for ninety-sixwell plates (Applied Biosystems). The primers of β-actin, superoxide dismutase 1 (*SOD*1), *CAT*, glutathione peroxidase 1 (*GPX*1), glutathione transferase (*GST*), glutathione reductase (*GR*), nuclear factor E2-related factor 2 (*Nrf*2), Kelch-like ECH-associated protein 1 (*Keap-*1) are shown in Table 2. The gene β-actin was used as an internal control. All the genes of each sample were repeated in triplicate. For each reaction, 5 μL of freshly SYBR® Premix Ex Taq™ II (Tli RNaseH Plus, 2×), 1 μL forward primers (4 mmol/L) and 1 μL reverse primers (4 mmol/L), 1 μL of cDNA and 2 μL nuclease-free water were added and made up to final volume of 10 μL. The PCR programme was as follow: a predegeneration at 95 °C for 10 min for one cycle, followed by denaturation at 95 °C for 5 s, annealing temperature at 60 °C for 45 s, extension temperature at 72 °C for 10 s for forty cycles. After amplification, the melting peaks of the amplification products were determined by melting curve which indicated only one expected amplification products had been generated. Melting curve conditions were as follows: 1 cycle of denaturation at 95 °C for 10 s and then 65 °C changed to 95 °C with a temperature change velocity of 0.5 °C/s. The standard curve of each gene was run in triplicate for obtaining reliable amplification efficiency values. The correlation coefficients of all the standard curves were > 0.99, and the amplification efficiency values were between 90 and 110%. Each primer pair used yielded a single peak in the melting curve and a single band with the expected size in agarose gel. The mean Ct values of duplicates of each sample were used for calculations. The relative gene expressions compared with the housekeeping gene b-actin were calculated by 2^-CT^ [31].

**Table 2:** Primers sequences used for quantitative RT-PCR^a^

Gene	Accession no.	Primer sequences (5′ - 3′)	Product Length, bp
β-actin	U07786.1	F:TCTGGCACCACACCTTCT	114
		R:TGATCTGGGTCATCTTCTCAC	
*SOD*1	AF396674.1	GATCAAGAGAGGCACGTTGGA	62
		GTGGCCACACCATCTTTGC	
*CAT*	NM_214	GGACGTGCAGCGCTTCA	52
		CCGCACCTGGGTGACATTA	
*GPX*1	NM_214201.1	GGCGGCGGGTTCGA	55
		CGCCATTCACCTCACACTTCT	
*GST*	Z69586.1	TCCCCACGGTGAAGAAGTTT	57
		CGTCAGTGGGAGGCTTCCT	
*GR*	AY368271.1	CAGTAGAGGTCAACGGGAAGAAGT	59
		GCCGCCTGTGGCAATC	
*Nrf*2	XM_003133500.5	GCCCCTGGAAGCGTTAAAC	67
		GGACTGTATCCCCAGAAGGTTGT	
*Keap-*1	NM_001114671.1	ACGACGTGGAGACAGAAACGT	56
		GCTTCGCCGATGCTTCA	

^a^*SOD*1, superoxide dismutase 1; *CAT*, catalase; *GPX*1, glutathione peroxidase 1; *GST*, glutathione transferase; *GR*, glutathione reductase; *Nrf*2, nuclear factor E2-related factor 2; *Keap-*1, Kelch-like ECH-associated protein 1

### Statistical analysis

All data were analysed using SPSS 20.0 software (SPSS, Inc.) by curve estimation model of regression procedure. The effect of PEO supplementation was determined by linear and quadratic effects in individual pig unit. All data were expressed as mean ± standard error. The significance level for all analyses was set at *P* < 0.05, with a trend of 0.05 ≤ *P* ≤ 0.01.

## Results

### Effect of PEO on growth performance

As indicated in Table 3, the BWG increased quadratically (*P* = 0.031), PEO200 increased the BWG 0.52 kg than CON. In addition, F:G also decreased linearly (*P* = 0.008) and quadratically (*P* = 0.007) as supplementation of PEO increased. PEO200 decreased 0.13 than CON in F: G.

**Table 3:** Growth performance of weaned pigs fed different levels of PEO^a^

Items^b^	CON	PEO50	PEO100	PEO200	*P*	
					Linear	Quadratic
IBW	9.25 ± 0.41	9.18 ± 0.24	9.16 ± 0.61	9.17 ± 0.49	0.977	0.294
FBW	15.53 ± 0.28	15.52 ± 0.20	15.55 ± 0.76	16.00 ± 0.54	0.922	0.895
BWG, kg	6.41 ± 0.55	6.33 ± 0.23	6.39 ± 0.71	6.83 ± 0.33	0.657	0.031
ADG, kg/d	0.45 ± 0.04	0.45 ± 0.02	0.46 ± 0.05	0.50 ± 0.03	0.114	0.099
ADFI, kg/d	0.67 ± 0.07	0.70 ± 0.03	0.67 ± 0.19	0.66 ± 0.04	0.770	0.829
F:G	1.47 ± 0.05	1.55 ± 0.07	1.43 ± 0.38	1.34 ± 0.09	0.008	0.007

Treatments: CON means basal diet; PEO50 means basal diet with 50 ppm PEO; PEO100 means basal diet with 100 ppm PEO; PEO200 means basal diet with 200 ppm PEO

^a^Values are means ± S.E, n = 6

^b^*IBW* initial body weight, *FBW* final body weight, *BWG* body weight gain, *ADG* average daily gain

### Effect of PEO on blood biochemical parameters and serum immunoglobulins

The effect of PEO supplementation on the level of serum TP, ALB, IgG, IgA and IgM were presented in Table 4. IgG increased linearly (*P* = 0.029) and IgM increased linearly (*P* = 0.003) and quadratically (*P* = 0.007) as supplementation of PEO increased. And then, supplementation of PEO tended quadratically (*P* = 0.098) increase IgM and linearly (*P* = 0.098) and quadratically (*P* = 0.070) increase ALB. Moreover, serum cholesterol was linearly (*P* = 0.039) as PEO supplementation.

**Table 4:** immune function of weaned pigs fed different levels of PEO^a^

Items^b^	CON	PEO50	PEO100	PEO200	*P*	
					Linear	Quadratic
TP, g/L	50.03 ± 1.69	50.66 ± 0.47	51.89 ± 0.64	51.91 ± 1.08	0.235	0.489
ALB, g/L	29.32 ± 0.82	28.98 ± 0.39	29.09 ± 0.35	30.90 ± 0.43	0.098	0.070
TG mmol/L	0.58 ± 0.06	0.44 ± 0.01	0.44 ± 0.07	0.43 ± 0.03	0.346	0.171
TC mmol/L	2.10 ± 0.04	2.29 ± 0.06	2.14 ± 0.06	1.95 ± 0.06	0.039	0.103
IgG, g/L	2.55 ± 0.25	2.42 ± 0.19	3.31 ± 0.43	3.84 ± 0.61	0.003	0.007
IgA, g/L	0.89 ± 0.05	0.89 ± 0.08	1.07 ± 0.08	1.14 ± 0.07	0.813	0.840
IgM, g/L	0.23 ± 0.03	0.25 ± 0.01	0.25 ± 0.02	0.23 ± 0.03	0.029	0.098

Treatments: CON means basal diet; PEO50 means basal diet with 50 ppm PEO; PEO100 means basal diet with 100 ppm PEO; PEO200 means basal diet with 200 ppm PEO

^a^Values are means ± S.E, n = 6

^b^*TP* total protein, *ALB* albumin, *TG* triglyceride, *TC* cholesterol

### Effect of PEO on serum antioxidant activity

There was a significant increase in serum GSH with increasing levels of PEO (linear, *P* < 0.01; quadratic, *P* < 0.01) (Table 5). Serum MDA increased linearly (*P* = 0.030) as supplementation of PEO increased.

**Table 5:** Antioxidant in serum of weaned pigs fed different levels of PEO^a^

Items^b^	CON	PEO50	PEO100	PEO200	*P*	
					Linear	Quadratic
CAT, U/mL	18.89 ± 1.26	19.62 ± 1.22	20.22 ± 0.52	20.18 ± 0.53	0.248	0.456
T-AOC, U/mL	2.10 ± 0.10	2.13 ± 0.12	2.16 ± 0.24	2.32 ± 0.13	0.451	0.642
MDA, nmol/mL	3.68 ± 0.40	3.21 ± 0.24	3.08 ± 0.47	2.67 ± 0.28	0.030	0.100
T-SOD, U/mL	18.15 ± 0.37	18.34 ± 0.18	18.67 ± 0.42	18.27 ± 0.13	0.721	0.388
GSH, mgGSH/L	3.12 ± 0.58	2.94 ± 0.38	3.98 ± 0.56	4.44 ± 0.39	< 0.01	< 0.01

total superoxide dismutase; GSH, glutathione

Treatments: CON means basal diet; PEO50 means basal diet with 50 ppm PEO; PEO100 means basal diet with 100 ppm PEO; PEO200 means basal diet with 200 ppm PEO

^a^Values are means ± S.E, *n* = 6

^b^*CAT* catalase, *T-AOC* total antioxidant capacity, *MDA* methane dicarboxylic aldehyde, *T-SOD* Total superoxide dismutase

### Effect of PEO on intestinal mucosal antioxidant activity

The effect of PEO supplementation on the level of intestinal mucosa TP, GSH, T-AOC, T-SOD, MDA and CAT were presented in Table 6. There was a significant increase in GSH with increasing levels of PEO in duodenal mucosa (linear, *P* = 0.007; quadratic, *P* = 0.029) and in ileal mucosa (linear, *P* = 0.011; quadratic, *P* = 0.032). MDA in jejunal mucosa decreased linearly (*P* = 0.042) as supplementation of PEO increased.

**Table 6:** Antioxidant in intestinal mucosa of weaned pigs fed different levels of PEO^a^

Items^b^	CON	PEO50	PEO100	PEO200	*P*	
					Linear	Quadratic
Duodenal mucosa						
TP, gprot/L	77.09 ± 7.06	78.38 ± 11.38	78.30 ± 6.59	76.29 ± 11.63	0.960	0.981
GSH, mgGSH/gprot	5.22 ± 0.93	5.73 ± 1.35	10.97 ± 0.80	12.02 ± 2.30	0.007	0.029
T-AOC, U/mgprot	0.21 ± 0.06	0.23 ± 0.02	0.28 ± 0.02	0.27 ± 0.01	0.157	0.369
T-SOD, U/mL	24.37 ± 1.83	24.79 ± 3.97	24.08 ± 1.71	24.90 ± 0.31	0.921	0.992
MDA, nmol/mL	36.93 ± 4. 50	26.64 ± 4.24	24.62 ± 6.15	19.37 ± 6.43	0.166	0.397
CAT, U/gprot	16.32 ± 2.15	17.73 ± 0.71	17.78 ± 1.83	18.55 ± 2.72	0.380	0.676
Jejunal mucosa						
TP, gprot/L	83.91 ± 10.41	85.10 ± 10.95	83.61 ± 10.58	84.48 ± 10.08	0.992	1.000
GSH, mgGSH/gprot	1.95 ± 0.47	2.48 ± 1.29	3.87 ± 1.35	2.80 ± 0.84	0.562	0.121
T-AOC, U/mgprot	0.23 ± 0.03	0.27 ± 0.01	0.28 ± 0.06	0.27 ± 0.07	0.381	0.600
T-SOD, U/mL	13.95 ± 0.97	16.16 ± 1.79	19.14 ± 3.02	20.12 ± 2.22	0.069	0.199
MDA, nmol/mL	37.60 ± 12.01	33.60 ± 3.27	32.90 ± 8.40	14.00 ± 2.79	0.042	0.076
CAT, U/gprot	4.63 ± 0.83	5.05 ± 0.39	5.35 ± 0.32	6.13 ± 1.13	0.254	0.528
Ileal mucosa						
TP, gprot/L	138.13 ± 6.15	160.16 ± 9.18	131.34 ± 10.15	149.74 ± 16.24	0.974	0.999
GSH, mgGSH/gprot	1.15 ± 0.11	1.98 ± 0.26	2.01 ± 0.29	2.35 ± 0.16	0.011	0.032
T-AOC, U/mgprot	0.14 ± 0.01	0.18 ± 0.03	0.19 ± 0.02	0.19 ± 0.03	0.126	0.210
T-SOD, U/mL	9.16 ± 0.80	9.11 ± 0.44	9.66 ± 0.17	9.68 ± 0.55	0.509	0.812
MDA, nmol/mL	91.41 ± 6.66	87.18 ± 13.15	83.04 ± 3.93	71.57 ± 4.29	0.246	0.506
CAT, U/gprot	5.36 ± 0.31	5.25 ± 0.83	6.64 ± 0.73	7.46 ± 1.03	0.114	0.279

Treatments: CON means basal diet; PEO50 means basal diet with 50 ppm PEO; PEO100 means basal diet with 100 ppm PEO; PEO200 means basal diet with 200 ppm PEO

^a^Values are means ± S.E, *n* = 6

^b^*TP* total protein, *GSH* glutathione, *CAT* catalase, *T-AOC* total antioxidant capacity, *MDA* methane dicarboxylic aldehyde, *T-SOD* total superoxide dismutase

### Effect of PEO on splenic, hepatic and pancreatic antioxidant activity

The levels of GSH, CAT, T-AOC, T-SOD and MDA in spleen, liver and pancreas were measured (Table 7). GSH in pancreas increased linearly (*P* = 0.007) and quadratically (*P* = 0.032) as supplementation of PEO increased. There was a decrease at 100 ppm (linear, *P* = 0.032) in MDA of pancreas with no further decrease at 200 ppm.

**Table 7:** Antioxidant in spleen, liver and pancreas of pigs fed different levels of PEO^a^

Items^b^	CON	PEO50	PEO100	PEO200	*P*	
					Linear	Quadratic
Spleen						
GSH, mgGSH/gprot	1.85 ± 0.51	1.65 ± 0.24	1.88 ± 0.04	2.19 ± 0.15	0.431	0.546
CAT, U/gprot	9.87 ± 0.71	9.33 ± 1.13	11.34 ± 0.68	10.49 ± 1.08	0.334	0.635
T-AOC, U/mgprot	0.21 ± 0.04	0.21 ± 0.05	0.22 ± 0.02	0.22 ± 0.02	0.812	0.963
T-SOD, U/mL	255.62 ± 10.52	247.22 ± 26.99	302.45 ± 8.38	291.02 ± 9.06	0.076	0.219
MDA, nmol/mL	146.00 ± 8.86	175.07 ± 26.70	128.41 ± 5.08	117.84 ± 7.44	0.132	0.190
Liver						
GSH, mgGSH/gprot	1.84 ± 0.30	1.35 ± 0.10	2.59 ± 0.30	2.53 ± 0.48	0.113	0.256
CAT, U/gprot	200.89 ± 19.50	204.58 ± 6.33	210.14 ± 19.42	206.30 ± 12.45	0.788	0.949
T-AOC, U/mgprot	0.52 ± 0.06	0.43 ± 0.11	0.49 ± 0.08	0.55 ± 0.13	0.489	0.238
T-SOD, U/mL	1127.18 ± 59.43	1132.51 ± 58.80	1161.75 ± 97.09	1200.82 ± 99.96	0.562	0.837
MDA, nmol/mL	273.32 ± 29.23	280.04 ± 26.55	266.00 ± 60.44	263.64 ± 21.62	0.835	0.974
Pancreas						
GSH, mgGSH/gprot	2.23 ± 0.30	2.41 ± 0.43	4.47 ± 0.66	4.52 ± 1.08	0.007	0.032
CAT, U/gprot	4.99 ± 0.79	5.14 ± 0.39	5.22 ± 0.12	6.40 ± 0.81	0.230	0.404
T-AOC, U/mgprot	0.14 ± 0.02	0.12 ± 0.02	0.13 ± 0.03	0.14 ± 0.01	0.906	0.804
T-SOD, U/mL	232.93 ± 16.47	207.09 ± 12.92	276.46 ± 25.64	257.91 ± 9.82	0.131	0.299
MDA, nmol/mL	45.52 ± 1.12	61.65 ± 9.02	28.64 ± 5.58	29.96 ± 3.40	0.032	0.068

aldehyde; T-SOD, total superoxide dismutase

Treatments: CON means basal diet; PEO50 means basal diet with 50 ppm PEO; PEO100 means basal diet with 100 ppm PEO; PEO200 means basal diet with 200 ppm PEO

^a^Values are means ± S.E, n = 6

^b^*GSH* glutathione, *CAT* catalase, *T-AOC* total antioxidant capacity, *MDA* methane dicarboxylic

### Effect of PEO on the expression of critical antioxidant-related genes

Gene expression of *GR*, *GST*, *GPX*1, *CAT*, *SOD*1, *Nrf*1 and *Keap*1 was measured (Table 8). Expression of *GST* in spleen increased linearly (*P* = 0.012) and quadratically (*P* = 0.007) as supplementation of PEO increased. There was a tendency to increase expression level of *Keap*1 with increasing levels of PEO in spleen (quadratic, *P* = 0.085). An increased in *GPX*1 (quadratic, *P* = 0.029), *CAT* (quadratic, *P* = 0.051) and *SOD1* (linear, *P* = 0.029) was observed in the spleen of pigs supplemented with 200 ppm PEO. Moreover, the expression of *GST* in liver increased quadratically (*P* = 0.049) as supplementation of PEO increased. However, the expression of *Nrf*2 in liver decreased linearly (*P* = 0.023) and quadratically (*P* = 0.029) as supplementation of PEO increased. Furthermore, there was no effect on the expression of *GR*, *GST*, *GPX*1, *CAT*, *SOD*1, *Nrf*1 and *Keap*1 in pancreas with increasing dietary inclusion level of PEO.

**Table 8:** The levels of the gene expression of antioxidant enzymes in spleen, liver and pancreas of weaned pigs fed diets with different levels of PEO^a^

Items^b^	CON	PEO50	PEO100	PEO200	*P*	
					Linear	Quadratic
Spleen						
*GR*	1.00 ± 0.18	0.79 ± 0.11	0.81 ± 0.04	1.12 ± 0.16	0.746	0.250
*GST*	1.00 ± 0.20	1.11 ± 0.25	1.12 ± 0.43	2.92 ± 0.42	0.012	0.007
*GPX*1	1.00 ± 0.21	0.81 ± 0.07	0.75 ± 0.07	1.42 ± 0.14	0.236	0.029
*CAT*	1.00 ± 0.15	0.63 ± 0.22	0.86 + 0.11	1.23 ± 0.07	0.366	0.051
*SOD*1	1.00 ± 0.18	0.76 ± 0.08	0.80 ± 0.08	1.31 ± 0.08	0.287	0.029
*Nrf*2	1.00 ± 0.21	0.94 ± 0.11	0.94 ± 0.07	1.13 ± 0.12	0.565	0.600
*Keap*1	1.00 ± 0.13	1.00 ± 0.07	1.04 ± 0.14	1.31 ± 0.07	0.085	0.129
*Liver*						
*GR*	1.00 ± 0.10	1.08 ± 0.27	1.18 ± 0.23	0.90 ± 0.15	0.867	0.602
*GST*	1.00 ± 0.22	0.71 ± 0.03	0.75 ± 0.18	1.16 ± 0.37	0.314	0.049
*GPX*1	1.00 ± 0.35	0.76 ± 0.30	0.94 ± 0.36	1.50 ± 0.43	0.421	0.456
*CAT*	1.00 ± 0.10	0.84 ± 0.19	0.81 ± 0.05	1.28 ± 0.14	0.373	0.114
*SOD*1	1.00 ± 0.13	1.16 ± 0.33	1.01 ± 0.15	1.08 ± 0.09	0.865	0.956
*Nrf*2	1.00 ± 0.07	1.07 ± 0.22	0.75 ± 0.27	0.16 ± 0.02	0.023	0.029
*Keap*1	1.00 ± 0.14	0.86 ± 0.17	0.93 ± 0.11	0.80 ± 0.11	0.354	0.658
*Pancreas*						
*GR*	1.00 ± 0.07	1.09 ± 0.22	1.15 ± 0.11	1.43 ± 0.39	0.342	0.628
*GST*	1.00 ± 0.31	0.94 ± 0.23	0.93 ± 0.24	1.06 ± 0.23	0.935	0.957
*GPX*1	1.00 ± 0.25	0.88 ± 0.04	1.34 ± 0.22	1.19 ± 0.27	0.402	0.714
*CAT*	1.00 ± 0.19	1.11 ± 0.23	1.07 ± 0.18	0.77 ± 0.13	0.502	0.569
*SOD*1	1.00 ± 0.10	0.66 ± 0.09	0.85 ± 0.16	1.03 ± 0.13	0.842	0.316
*Nrf*2	1.00 ± 0.12	0.91 ± 0.16	0.96 ± 0.14	0.95 ± 0.19	0.858	0.975
*Keap*1	1.00 ± 0.21	0.96 ± 0.08	1.24 ± 0.18	1.23 ± 0.16	0.326	0.629

Treatments: CON means basal diet; PEO50 means basal diet with 50 ppm PEO; PEO100 means basal diet with 100 ppm PEO; PEO200 means basal diet with 200 ppm PEO

^a^Values are means ± S.E, n = 6

^b^*SOD*1, superoxide dismutase 1; *CAT*, catalase; *GPX*1,glutathione peroxidase 1; *GST*, glutathione transferase; *GR*, glutathione reductase; *Nrf*2, nuclear factor E2-related factor 2; *Keap-*1, Kelch-like ECH-associated protein 1

## Discussion

Weaning stress syndrome was an inevitable problem, which could lead weaning stress, oxidative stress, and adversely affects intestinal health, leads to diarrhea, and reduces growth and immunity in pigs [2–4]. Oxidative stress is caused by excess oxidative radicals, including reactive oxygen species, which damage DNA, bio-membrane lipids, and proteins, and also impair tissue function [32, 33]. This may be the reason that weaning stress syndrome reduces growth performance and economic benefit in pig production. Thymol and cinnamaldehyde are concentrated hydrophobic liquids containing volatile aromatic compounds extracted from plants, which have unique chemical structures (Fig. 1) and could be used as natural antioxidants [11, 34].

In this study, dietary supplementation of PEO at a dose of 200 ppm quadratically improved BWG of pigs after weaning. As well as, ADG tended to increase quadratically as 200 ppm PEO supplementation increased. Because of there was no effect on average daily feed intake (ADFI) with the increasing PEO addition. So the most important reason for growth performance improvement was feed efficiency. F:G decreased linearly and quadratically as PEO supplementation and 200 ppm PEO supplementation obtained the best F:G. In agreement with our results, previous studies have documented similar positive effects of PEO supplementation on growth performance in weaned pigs [17, 24]. The improvement in nutrient absorption may be partly explained by increased secretions of saliva, bile and enhanced enzyme activity [35–38].

Moreover, PEO supplementation has been reported to improve the immune status of pigs after weaning, as indicated by increased serum immunoglobulin levels [15, 17]. Our results indicated that PEO supplementation increased the levels of ALB, IgA, and IgG in serum, and 200 ppm PEO addition got the highest levels. It is well known that IgG offers newborn pigs extended systemic protection, while IgA and IgM offer transient luminal protection [39]. During the first few days after weaning, piglets often experience low feed intake or starvation issues that can represent a major challenge for the producer. Therefore, the administration of 200 mg/kg PEO to piglets during this time probably improve their immune status and prevent oxidative stress.

Oxidative stress is caused by excess oxidative radicals, including reactive oxygen species (ROS) and reactive nitrogen species (RNS), which damage DNA, bio-membrane lipids, proteins, and other macromolecules [32]. However, excess oxidative radicals can be eliminated by antioxidants, including nonenzymatic components and a series of enzymes. Within the enzymatic antioxidant system, superoxide dismutase and glutathione peroxidase are the most important compounds [21], working together to detoxify superoxide anions and hydrogen peroxide in cells [33]. Superoxide dismutase catalyzes superoxide anions to produce hydrogen peroxide and molecular oxygen. Glutathione peroxidase normally converts H_2_O_2_ to water [21]. The capabilities of the non-enzymatic antioxidant defense system are often measured as the total antioxidant capacity [40]. Furthermore, the level of malondialdehyde, a major product of lipid peroxidation, is an effective marker of oxidative stress in sepsis [21].

In the present study, we measured GSH, CAT, MDA, and T-SOD levels, as well as T-AOC, in serum, intestinal mucosa, spleen, liver, and pancreas. Present results indicated that PEO supplementation significantly increased GSH levels in serum, duodenal and ileal mucosa and pancreas, T-SOD activity in jejunal mucosa and spleen, and significantly decreased MDA levels in serum, jejunal mucosa and pancreas. The results of this study were consistent with previous reports that T-AOC was improved and plasma MDA level was decreased in pigs receiving supplemental PEO [15, 41, 42]. Previous study have confirmed that PEO could save the depletion of SOD and GSH-Px or enhance their capabilities [21]. The increased GSH and decreased MDA levels in serum indicated that whole-body antioxidant status was improved, and lipid peroxidation was reduced. In addition, the reduced MDA levels and improvements in GSH levels and T-SOD activity suggested that PEO could enhance both the non-enzymatic and enzymatic reactions of the antioxidant defense system. Various natural antioxidant extracts have been used to protect pigs from weaning stress in intensive pig production [1]. Our results suggests that dietary PEO supplementation may reduce oxidative stress and have the potential to ameliorate the adverse effects of early weaning syndrome.

In order to elucidate the mechanism of PEO antioxidant activity in weaned pigs, we measured the expression of *SOD*1, *CAT*, *GPX*1, *GST*, *GR*, *Nrf*2, and *Keap-*1 in the spleen, liver, and pancreas. *Keap-*1 is the cytosolic protein with which the transcription factor *Nrf*2 is associated, and which functions to protect against oxidative stress. Oxidative stress modifies *Keap-*1 at redox-sensitive SH groups, leading to the liberation and nuclear translocation of *Nrf*2. Subsequently, *Nrf*2 binds to the ARE promoter sequence of antioxidant enzymes. In this way, *Nrf*2 coordinates cytoplasmic responses to oxidative stress [43]. A previous study reported that essential oils increased the mRNA expression of jejunal *GPx*1 and *SOD*1, ileal *GST*, and colonic *GPX*1, *SOD*1, and *Keap-*1 [44]. Similarly, the present results indicated that PEO supplementation upregulated the expression of *GST*, *GPX*, *CAT*, and *SOD*1 in the spleen and the expression of *GST* and *Nrf*2 in the liver. It can be speculated that the different terpene compounds in PEO can modify Keap 1 at sensor –SH groups through chemical reactions [45]. Oxidative stress modifies Keap1 at redox-sensitive -SH groups, which leads to Nrf2 liberation and its nuclear translocation. Subsequently, Nrf2 binds to the ARE promoter sequence of antioxidant enzymes. In this way, Nrf2 coordinates cytoplasmic responses to oxidative stress [43]. The changes in the expression of genes associated with oxidation and antioxidant compounds may explain the findings in serum and tissue samples. The spleen is a vital part of the immune system and the liver is important in detoxification and metabolism in pigs [46, 47]. The improved splenic and hepatic antioxidant capacities may be reflected in the immunity and health of weaned pigs. Although the PEO used in our study has been confirmed to improve the antioxidant capacities of serum and other tissues, the molecular mechanisms through which PEO impacts the upstream *Nrf*2 pathways and then modulates antioxidant capacity remains to be further investigated.

## Conclusions

In conclusion, our results suggest that dietary PEO supplementation improved growth performance, immune function, and antioxidant status in weaned pigs. The benefits observed may be mediated by upregulation of antioxidant-related genes in the spleen and liver. And that supplementation of the PEO preparation at the levels of 200 mg/kg diet would seem to be economically feasible. This study not only provides new insights into the role of PEO in improving growth performance, immune function, and antioxidant capacity in weaned pigs, but also reveals a potential candidate to replace the antibiotics conventionally used in the livestock industry.

## Abbreviations

ALB: Albumin
CAT: Catalase
GPX1: Glutathione peroxidase 1
GR: Glutathione reductase
GSH: Glutathione
GST: Glutathione transferase
Keap-1: Kelch-like ECH-associated protein 1
MDA: Methane dicarboxylic aldehyde
Nrf2: Nuclear factor E2-related factor 2
PEO: Plant essential oil
*SOD*1: Superoxide dismutase 1
T-AOC: Total antioxidant capacity
TP: Total protein
T-SOD: Total superoxide dismutase
